# Ecological Exposure History Shapes Giraffe Vigilance Responses to Anthropogenic Noise: A Multisite Playback Experiment

**DOI:** 10.1002/ece3.72763

**Published:** 2025-12-17

**Authors:** Kaitlyn Taylor, Sean van der Merwe, Francois Deacon, Andri Grobbelaar, Anton Baotic

**Affiliations:** ^1^ Department of Animal Sciences, Faculty of Natural and Agricultural Sciences University of the Free State Bloemfontein South Africa; ^2^ Anne Innis Dagg Foundation Toronto Canada; ^3^ Department of Mathematical Statistics and Actuarial Science University of the Free State Bloemfontein South Africa; ^4^ Acoustics Research Institute Austrian Academy of Sciences Vienna Austria

**Keywords:** anthropogenic noise, bioacoustics, giraffe, playback experiment, sensory ecology, vigilance behavior

## Abstract

Anthropogenic noise is an increasingly pervasive environmental stressor for wildlife, yet its behavioral impacts on large mammals remain poorly understood. Across the genus *Giraffa,* multiple giraffe species face varying degrees of conservation condern under IUCN criteria due to rapid habitat modification and increasing human disturbance, yet the behavioral consequences of anthropogenic noise exposure have not been experimentally tested. Because vigilance is a key anti‐predator response in large herbivores and is highly sensitive to disturbance, we used it as a focal metric to assess giraffe reactions to noise. We conducted multisite playback experiments across three reserves in the Free State Province, South Africa, that differed in human‐exposure levels. Free‐roaming giraffes were presented with three anthropogenic sound stimuli (drone, vehicle, people talking) and a natural control stimulus (ring‐necked dove). Vigilance responses were video‐recorded, quantified, and analyzed using mixed‐effects models. Giraffes at the low‐exposure site showed markedly stronger vigilance responses to anthropogenic than to the natural control, whereas responses at high‐exposure sites were weaker and less differentiated across stimuli. These site‐specific patterns indicate that ecological exposure history modulates giraffe responsiveness to noise. Across the full dataset, anthropogenic sounds consistently elicited stronger vigilance responses than the control stimulus, demonstrating that noise alone, independent of visual cues, can alter natural behavior in free‐roaming giraffes. Collectively, these findings highlight the importance of incorporating acoustic disturbance into environmental impact assessments and protected‐area management. More broadly, the results contribute to understanding how wildlife perceive and respond to human‐generated noise and emphasize the need to integrate soundscape considerations into conservation planning as anthropogenic noise continues to expand across savanna ecosystems.

## Graphical abstract

Understanding how animals respond to anthropogenic disturbance is essential for predicting the impacts of human activity on wildlife behavior and persistence. As noise increasingly alters sensory landscapes across ecosystems, it can influence how animals detect threats and assess risk. The Southern giraffe served as the focal species of a multisite playback experiment, allowing comparisons of behavioral responses to anthropogenic noise across populations with different exposure histories.

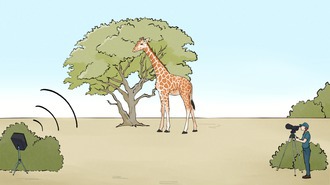

## 1 Introduction

Southern Africa, including South Africa, is undergoing rapid environmental transformation driven by population growth, agricultural expansion, infrastructure development, and tourism‐related land use. These changes have led to habitat fragmentation, altered ecological processes, and increased human‐wildlife interaction (Venter et al. 2016; Lindsey et al. 2022). Giraffes (*Giraffa* spp.), which inhabit many of these transforming landscapes, are increasingly confronted with anthropogenic disturbances, including visual and acoustic cues associated with human activity, as a result of expanding human settlement, infrastructure, and tourism development (Shannon et al. 2016; Gaynor et al. 2018; Bond et al. 2023; Gašparová et al. 2023; Vukelić et al. 2025). In areas of higher human activity, giraffes have been shown to occupy smaller home ranges, exhibit reduced daily movement rates (Brown et al. 2023), and form more fragmented and weaker social associations compared to populations in less disturbed environments (Bond, König, Lee, et al. 2021).

Among the various forms of human disturbance encountered by giraffes and other large herbivores, anthropogenic noise has received increasing attention as a factor capable of altering wildlife behavior, health and habitat use (Kunc and Schmidt 2019; Muller et al. 2019; Bond et al. 2023; Brown et al. 2023). Human‐generated sounds (such as those produced by vehicles, drones, machinery, and voices) can alter the acoustic environment that animals use for communication, risk assessment, and detection of other biologically relevant cues, such as the sounds of approaching predators (Blickley and Patricelli 2010; Francis and Barber 2013; Rosa et al. 2015). Behavioral responses to anthropogenic noise may include changes in temporal patterns, altered movement and space‐use decisions such as avoiding noisy areas, reduced foraging efficiency, increased vigilance, and disruptions to parental care, communication, or territorial interaction, all of which can scale up to affect reproductive success, fitness and population dynamics (Blickley and Patricelli 2010; Halfwerk et al. 2011; Francis and Barber 2013; Gill et al. 2014; Rosa et al. 2015; Phillips and Derryberry 2018; Tennessen et al. 2024). Such adjustments often occur because noise interferes with the detection of biologically relevant cues and alters the balance between food intake and predator avoidance, which reflects the classic starvation‐predation trade‐off described in behavioral ecology (Lima and Dill 1990; Frid and Dill 2002; Brown et al. 2012) and can lead animals to increase vigilance or avoidance at the expense of feeding opportunities (Francis and Barber 2013; Kok et al. 2023). In regions where giraffes coexist with expanding tourism and infrastructure, acoustic disturbance represents a potentially important but underexplored factor influencing their behavior, particularly their vigilance.

Giraffes are highly vigilant herbivores that rely on their height to visually monitor their surroundings. In most mammals, vigilance typically declines as group size increases, reflecting the classic ‘many eyes’ effect (Roberts 1996; Hunter and Skinner 1998; Creel et al. 2014; Beauchamp et al. 2021). However, giraffes appear to deviate from this pattern. Cameron and du Toit (2005) found that group size had little effect on vigilance behavior in giraffes. Instead, scanning behavior was more influenced by social factors, such as which individuals were present and their proximity. This aligns with broader findings that social context, the strength and stability of social associations, and the structure of local social networks (especially in anthropogenically disturbed landscapes) can all shape risk perception and anti‐predator behavior (Bond, König, Lee, et al. 2021; Bond, Lee, Farine, et al. 2021; Bond, Lee, Ozgul, et al. 2021). Sex differences have also been reported, with females tending to initiate vigilance more frequently and males engaging in longer vigilance bouts, resulting in similar overall vigilance effort (Cameron and du Toit 2005). Further, a recent study on Maasai giraffes found that, while the proportion of vigilant individuals decreased with group size, the total number of vigilant individuals, suggesting a balance between individual and group‐level risk management (Marealle et al. 2020). Vigilance was heightened in areas with increased poaching pressure, even when giraffes themselves were not directly targeted, which suggested that anthropogenic threats and disturbance amplified risk perception and vigilance responses. Recent experimental work has shown that giraffe vigilance is flexible and context‐dependent, and that it is shaped by ecological experience. Both the perception of risk and the magnitude of vigilance responses to predator cues and heterospecific alarm calls are modulated by a giraffe's prior exposure to predators and disturbance. Together, these findings underscore the key role of ecological experience in shaping anti‐predator behavior (Bond, König, Lee, et al. 2021; Bond et al. 2023; Baotic and Szipl 2025a, 2025b).

Multiple studies have shown that giraffe behavior, space use, group composition, and stress physiology vary with ecological conditions, predation risk, and anthropogenic factors such as proximity to humans, livestock, and land‐use change (Muller et al. 2019; Bond, König, Lee, et al. 2021; Scheijen et al. 2021; Brown et al. 2023; Deacon et al. 2024; Hejcmanová et al. 2024; Vukelić et al. 2025). Long‐term monitoring indicates that, with effective conservation and community partnerships, giraffes can persist and even recover in densely populated agricultural areas outside protected reserves (Vukelić et al. 2025). Within this flexible social system, fission‐fusion acts as the proximate way these drivers are expressed in day‐to‐day behavior: food limitation and the dry season have been associated with smaller groups, predation risk, especially in groups with calves, shifts habitat use toward bushland and smaller units, and proximity to humans fragments social associations and redistributes groups relative to towns and bomas (Bercovitch and Berry 2010; Bond et al. 2019; Muller et al. 2019; Bond, König, Lee, et al. 2021). Together, these changes reconfigure who associates with whom and when, which likely modulates vigilance coordination and other responses to disturbance. How giraffes respond to acute sensory disturbances, particularly anthropogenic noise, remains poorly understood. Recent work has examined responses to drones in both wild (Bennitt et al. 2019; vanVuuren et al. 2023) and captive (Mesquita et al. 2022) settings, showing clear alert, vigilance, and movement responses that were stronger at lower flight altitudes and diminished with repeated passes, consistent with rapid habituation to unmanned aerial vehicle (UAV) (vanVuuren et al. 2023). Across taxa, UAV responses are shaped by operational parameters, including flight altitude, proximity and approach, speed, noise output, and prior exposure history (Afridi et al. 2025). Yet these drone studies did not separate the visual and acoustic pathways, as the drone remained visible during the trials. Since animals are typically exposed to both visual and acoustic cues simultaneously, disentangling the role of sound requires controlled experiments that present sound without a visual stimulus (Rosa et al. 2015). Animals rely heavily on acoustic cues to evaluate risk and adjust vigilance accordingly. Anthropogenic noise can therefore function as a salient but unfamiliar cue that alters perceived environmental risk. Emerging evidence indicates that animals assess human‐generated sounds using sensory and risk‐assessment mechanisms similar to those engaged during natural threat evaluation, with novelty, unpredictability and prior ecological experience strongly shaping behavioral responses (Kok et al. 2023). Playback experiments therefore provide a critical link for testing noise‐specific effects in giraffes, including sensitivity to sound alone, the role of prior exposure, and comparisons with non‐disturbance controls.

Playback experiments have been widely used to assess animal responses to noise in a controlled and ecologically relevant way (McGregor et al. 1992; Douglas and Mennill 2010; Rosa et al. 2015; Shannon et al. 2016). This method has proven valuable in studying and comprehending behavioral responses to anthropogenic noise across a variety of taxa (Radford et al. 2016; Martin et al. 2022; Petric and Kalcounis‐Rueppell 2023; Sørensen et al. 2023). For example, in a recent playback study conducted in Greater Kruger National Park in South Africa, giraffes exhibited stronger behavioral responses to human voices than to lion vocalizations, suggesting that they may perceive human‐generated sound as a particular threat (Zanette et al. 2023).

Building on this framework, we tested how wild giraffes respond to different types of human‐generated noise. We conducted playback experiments across three nature reserves in South Africa that vary in daily exposure to human activity, allowing us to assess both stimulus‐specific and context‐dependent variation in responses. We use vigilance as a measurable and ecologically meaningful response variable to evaluate how giraffes perceive and assess acoustic risk in their environment. By experimentally isolating sound in the absence of visual cues, this study provides a direct test of how giraffes evaluate anthropogenic noise across a gradient of human disturbance. This design allows us to identify both stimulus‐specific and context‐dependent components of noise perception, and to assess how ecological exposure history shapes vigilance responses. This study is, to our knowledge, the first to systematically test noise‐related vigilance responses in free‐ranging giraffes using controlled playback experiments. We predicted that: (1) giraffes would exhibit increased vigilance to anthropogenic versus natural sounds, and (2) individuals from low‐exposure sites would respond more strongly than those from high‐exposure sites. Together, these predictions allow us to determine how anthropogenic noise shapes vigilance behavior in a species inhabiting increasingly human‐influenced savanna ecosystems.

## 2 Methods

### 2.1 Study Site

This study was conducted at three different game reserves within the Free State Province of South Africa with data collected from July 2023 to June 2024 during daylight hours (08:00–17:00 local time). No large predators were present during the study period. The reserves were selected to compare behavioral responses of Southern giraffes ( *Giraffa giraffa* ) to anthropogenic noise under different levels of habituation and environmental exposure (summarized in Table 1). We classified human exposure a priori using three criteria: (i) frequency of direct human presence within giraffe core areas (pedestrians, vehicles on internal roads, maintenance, events), (ii) public access regime, and (iii) chronic background noise from adjacent infrastructure.

**TABLE 1:** Overview of study sites. Descriptive information of the three study sites located in the Free State Province, South Africa, where Southern giraffes ( *Giraffa giraffa* ) playback trials were conducted between 2023 and 2024. Variables include reserve size (in hectares), vegetation types with their respective classification codes (Mucina and Rutherford 2006), giraffe population size, the demographic composition based on number of individuals that are male/female and sub‐adult/adult with the age classes following criteria established by Muller et al. 2022, level of daily human interaction, and presence of natural predators during the study period.

Study site	Size	Vegetation unit (Code)	*N* individuals tested	Female		Male		Daily exposure with humans^a^	Predators present
				Sub‐adult	Adult	Sub‐adult	Adult		
Franklin Game Reserve	250	Winburg Grassy Shrubland (Gh 7)	4	1	1	0	2	High	None
Weltevreden Game Lodge	500	Winburg Grassy Shrubland (Gh 7), Bloemfontein Karroid Shrubland (Gh 8), and Highveld Alluvial Vegetation (Aza 5)	10	2	4	0	4	Moderate	None
Amanzi Private Game Reserve	7116	Winburg Grassy Shrubland (Gh 7), and Vaal‐Vet Sandy Grassland (Gh 10)	19	0	8	7	4	Limited	None

^a^ The human activity considers the number of times the giraffes encounter people through game drives, people walking, and location proximity to human settlement.

Franklin Game Reserve (FGR). FGR lies within the city limits of Bloemfontein, is surrounded by urban development, and is a public reserve. The reserve is approximately 250 ha in size and is characterized by a variety of habitats ranging from shrubland to open grasslands (Mucina and Rutherford 2006). Giraffes at FGR are highly habituated to human presence, encountering pedestrians, vehicles, and reserve maintenance activities on a daily basis. This frequent direct exposure, together with continuous road and pedestrian activity within and around the reserve, supports its classification as a high‐exposure site.

Weltevreden Game Lodge (WGL) is located 35 km outside the city limits of Bloemfontein and is approximately 500 ha in size. The reserve includes karroid shrubveld, dry grassland, and riparian thickets along seasonal drainage lines (Mucina and Rutherford 2006). Ecotourism activities include game drives, recreational hunting, and special lodge events. Although tourist activity is moderate and access is controlled, the boundary runs directly alongside the national N1 highway, exposing giraffes to persistent road noise. Animals are moderately habituated, and we therefore classified WGL as moderate exposure because direct encounters on internal roads are intermittent, but chronic highway noise is present at the boundary.

Amanzi Private Game Reserve (APGR). APGR is more than 80 km from Bloemfontein and covers approximately 500 ha, with open grasslands, hills and slopes, and shrublands (Haddad and Butler 2018). This reserve also offers guided game drives, recreational hunting, and occasionally large events. Ecotourism activities are restricted to designated routes, and giraffes elsewhere in the reserve rarely encounter people. Due to its remote location, APGR experiences low levels of background noise from external infrastructure and infrequent direct human presence, and was therefore classified as a low‐exposure site.

**FIGURE 1:**
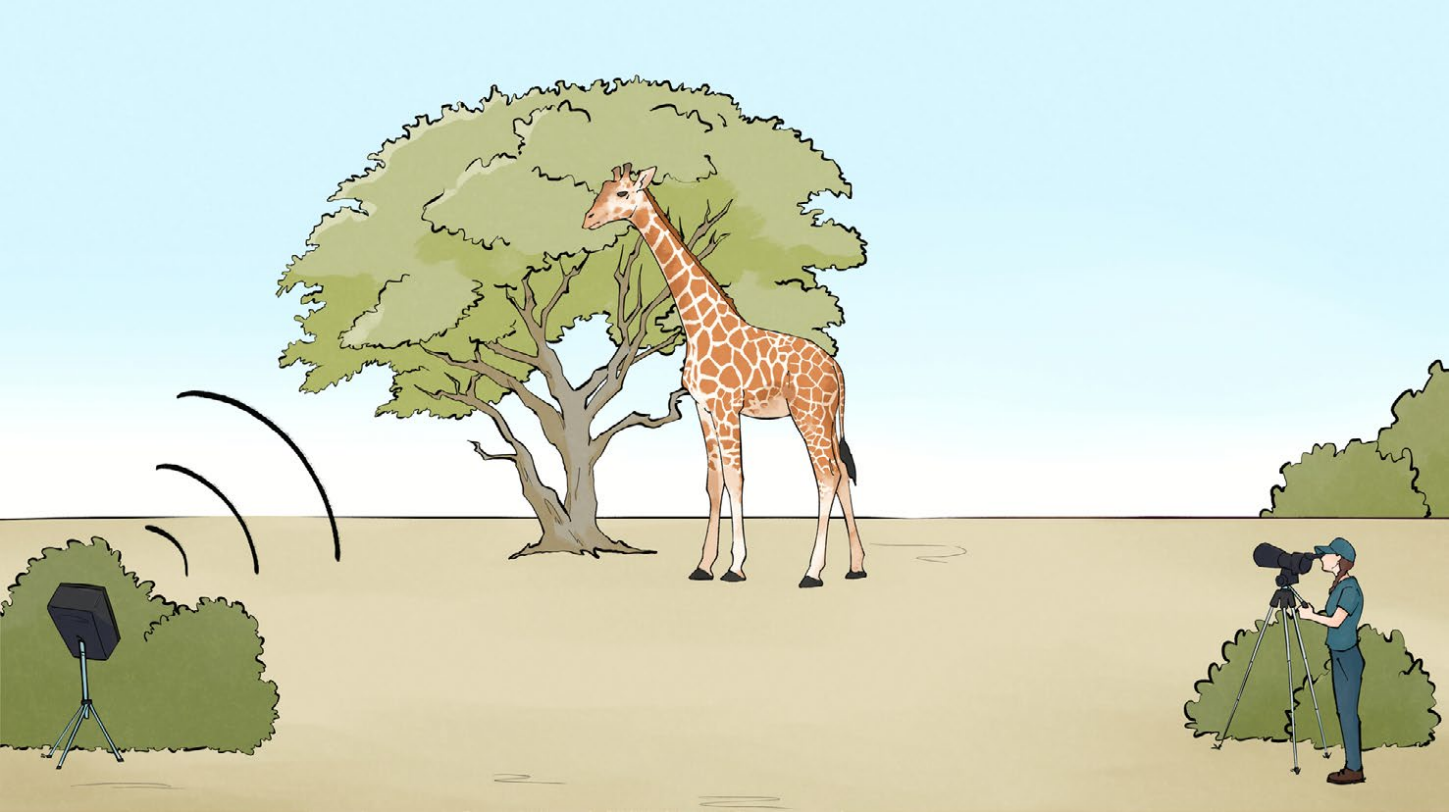
Illustration of the experimental playback setup used for behavioral observations of giraffes at the three study sites in the Free State Province, South Africa. During trials, a focal giraffe was first located from a vehicle and then approached on foot. A JBL‐EON Compact loudspeaker was placed on a tripod approximately 1 m above the ground and concealed behind dense vegetation to prevent visual detection. The observer stood parallel to the loudspeaker at a distance of 15–55 m, while the loudspeaker was positioned 30–100 m from the giraffe. Playbacks consisted of one anthropogenic test stimulus (drone, vehicle, or people talking) and one natural control stimulus (ring‐necked dove coo calls), presented in randomized order.

Across all three study sites, 36 giraffes were observed between July 2023 and June 2024. A total of 241 videos yielded 527 individual giraffe observations. Each giraffe was presented with a control stimulus (ring‐necked dove) and one anthropogenic test stimulus (drone, vehicle, or human voice). The playback setup is illustrated in Figure 1.

### 2.2 Playback Stimuli: Collection and Acoustic Characterization

**FIGURE 2:**
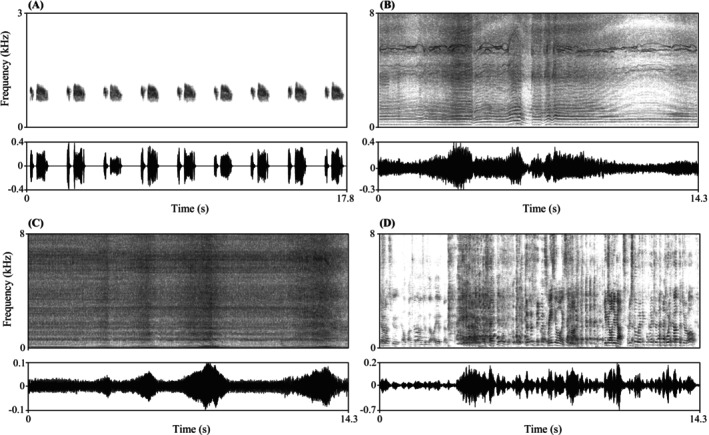
Spectrograms and oscillograms of the playback stimuli. (A) ring‐necked dove ( *Streptopelia capicola* ) stimulus composed of nine coo calls, (B) drone noise sequence, (C) vehicle engine noise, and (D) people talking. Spectrograms were generated in Praat using a fast Fourier transform (FFT) method with a dynamic range of 65.0 dB. Window length was set to 0.03 s for the vehicle and drone stimulus and 0.04 s for the dove and people talking stimulus.

Three types of anthropogenic noise test stimuli were selected for playback to reflect common sound sources associated with human activity in southern African savanna landscapes: (1) human voices, (2) vehicle engine sounds, and (3) drone noise. Human‐voice exemplars were unfamiliar to the study animals. A naturalistic non‐alarm control stimulus, the ‘coo’ call of the ring‐necked dove ( *Streptopelia capicola* ) (Slabbekoorn et al. 1999), was also included to serve as a biologically neutral baseline. Ring‐necked doves are common at all three reserves, so this control represented a familiar, non‐novel background sound. We treated vehicle engines, drone noise, and human voices as anthropogenic acoustic cues. While vehicle and drone sounds commonly co‐occur with human activity, our aim was to test responses to sound itself. The playback design therefore isolates acoustic effects from visual cues or explicit attribution to humans. Representative spectrograms of all stimulus types are shown in Figure 2.

The four playback stimuli also differed naturally in their acoustic structure. Dove coo calls are periodic, tonal signals with relatively high harmonic structure (Slabbekoorn et al. 1999; Shieh et al. 2016), whereas the anthropogenic sounds (vehicle engines, drones, and human voices) are spectrally more complex. Vehicle traffic noise is typically broadband and dominated by low frequencies (Shannon et al. 2016). Drone acoustic emissions span a wide frequency range from 20 Hz to 16.25 kHz (Stepien et al. 2024), and therefore overlap broadly with the auditory sensitivity of many terrestrial (Stansbury et al. 2025) and marine vertebrates (Dudzinski et al. 2025). Human speech is acoustically complex, containing both voiced (harmonic) components and unvoiced aperiodic elements (Lindblom and Sundberg 2007). Beyond these spectral properties, human vocalizations are ecologically relevant because they reliably signal the presence of humans (potential predators in many contexts) and can elicit vigilance or avoidance in large mammals (Suraci et al. 2019).

Drone sounds were recorded using a DJI Phantom 4 drone operated at 1 m above the recording equipment, flying randomly within a 5‐m radius. Vehicle engine recordings were captured by driving a Great Wall Motors (GWM) pick‐up truck in irregular patterns within a similar radius, alternating between idling and low‐speed movement. Human voice recordings consisted of five adult speakers (three males, two females) engaging in casual conversation in English and Afrikaans. To avoid familiarity effects, none of the recorded speakers were observers, reserve staff, guides, or frequent visitors at any study site, so the human‐voice exemplars were unfamiliar to all focal giraffes. These recordings were made from a distance of 5 m. All anthropogenic stimuli were recorded using a Sound Devices MixPre‐3 II recorder (frequency response: 10 Hz–80 kHz, 44.1‐kHz sample rate, 16‐bit resolution) connected to a Neumann KM 183 omnidirectional microphone fitted with a Rycote Cyclone windscreen to minimize wind noise. Ring‐necked dove coo calls were recorded using a Tascam DR‐07X handheld recorder (same sample rate and resolution). Recordings were made opportunistically from approximately 10 m away, targeting vocalizing individuals between dusk and dawn in an urban garden in Bloemfontein. The microphone was directed toward the bird's beak to ensure minimal background interference and optimal signal clarity. All audio files were stored as uncompressed WAV files at 44.1‐kHz/16‐bit resolution and screened for signal quality and the absence of friction sounds.

### 2.3 Editing Playback Stimuli

All recordings were edited and processed using Audacity (v3.3.2) and Praat (v6.3.09) to ensure acoustic consistency and comparability across stimulus types. Each file underwent a multi‐step procedure involving noise reduction, frequency filtering, amplitude normalization, and sequence construction. A one‐second segment of ambient noise preceding each recording was extracted to create a noise profile, which was used in Audacity's spectral noise reduction function (2 dB per pass, default sensitivity, and smoothing parameters). High‐pass and low‐pass filters were applied to limit excess frequency ranges. Dove calls were filtered with a high‐pass cut off at 450 Hz, while anthropogenic stimuli were filtered at 20 Hz. A low‐pass filter at 1.3 kHz was applied to all dove coo calls to eliminate high‐frequency background noise. A final 100 Hz smoothing filter was applied to reduce spectral artifacts and ensure acoustic clarity. Following noise filtering, all files were peak‐normalized to −1.0 dB using Audacity. Final amplitude levels were verified using a MAC AFRIC Digital Sound Level Meter.

In total, eight unique 2‐min playback files were constructed for each stimulus type. For dove calls, nine high‐quality coo vocalizations were extracted from each recording, separated by 1 s of silence, and randomly re‐ordered into four sequences of approximately 18 s each. These sequences were then combined with randomly selected inter‐sequence silent intervals of 12–26 s to create 2‐min playback files. Anthropogenic stimuli were edited into eight independent exemplars per stimulus type, each with a duration of two minutes. The dove control comprised naturalistic‐sounding coo bouts separated by silence to mimic typical calling, whereas the anthropogenic exemplars retained the natural temporal pattern of each source (drone rotor noise, vehicle engine noise variability, conversational speech with natural pauses). No fade‐in or fade‐out amplitude envelopes were applied at the onset and offset of the playback stimuli; each track began at the calibrated level from onset. The eight exemplars per category were rotated across trials. Usage per exemplar ranged from 17 to 41 for doves (total 239 uses), 6–19 for human voices (total 87), 6–16 for vehicles (total 76), and 2–17 for drones (total 74). Detailed per‐exemplar counts are provided in Table S1. All playback files were screened to avoid clipping or editing artifacts and saved as mono WAV files (48 kHz, 16‐bit). The complete stimulus set consisted of 79 playback packages, each comprising one anthropogenic sound file and one dove control, with randomized presentation order to prevent sequence bias.

### 2.4 Sound Pressure Level

To determine sound pressure levels (SPL) per stimulus type, we used published SPL values measured at 1 m for human speech (Pearsons et al. 1977) and for ring‐necked dove calls (De Kort et al. 2002; Beckers et al. 2003), supplemented by reference field measurements from our own playback exemplars. Using a MAC AFRIC digital sound level meter positioned 1 m in front of a JBL‐EON Compact loudspeaker, we documented the maximum and minimum output across eight exemplars of each stimulus type, which yielded reference levels of 55 dB for drone noise and 68 dB for vehicle engine sounds.

To ensure ecologically realistic playback levels, amplitude calibration for each stimulus type was guided by these reference SPLs. The ‘playback values’ reported in Table 2 (e.g., 68–78 dB for drones) reflect natural within‐exemplar amplitude fluctuation within a single sound file (such as propeller acceleration, engine rhythm, or speech dynamics) rather than differences between exemplars or playback events. Prior to field sessions, playback amplitudes were adjusted to match the reference levels using the loudspeaker's built‐in visual indicators, and the output was verified with the sound‐level meter to ensure that the selected volume setting produced SPLs within the target range. Playback calibration was performed once before data collection by placing a sound level meter 1 m in front of the JBL‐EON Compact loudspeaker and adjusting the output to the reference SPL. After this initial calibration, the loudspeaker settings remained unchanged throughout all playback trials. A tabular overview is provided in Table 2.

**TABLE 2:** Sound pressure levels (SPL in decibels, dB) of the four different playback stimulus types: Drone, vehicle, people talking and ring‐necked dove. Reference values were obtained from literature and field observations. Playback values represent the measured range within exemplars during calibration. Mean playback SPLs (midpoints) indicate the approximate average amplitude of each sound type used in the study.

Measurement type	SPL per stimulus type in dB			
	Drone	Vehicle	People talking	Ring‐necked dove
Reference values	68^a^	55^a^	55–68^b^	65–75^c^, ^d^
Playback values	68–78	55–64	58–68	65–73
Mean playback SPLs	73	60	63	69

^a^ Calculated based on field experiments.

^b^ Pearsons et al. (1977).

^c^ Beckers et al. (2003).

^d^ De Kort et al. (2002).

Although playback levels were calibrated once before data collection, SPLs were periodically verified in the field before representative playback sessions to confirm that output levels remained consistent with naturally occurring sources. Playback levels during trials ranged from 68 to 78 dB for drones, 55–64 dB for vehicles, 58–68 dB for human voices, and 65–75 dB for dove calls. The resulting mean playback SPLs were approximately 73 dB for drones, 60 dB for vehicles, 63 dB for human voices, and 69 dB for dove calls, all of which fall within the natural amplitude ranges reported for these stimulus types under field conditions. While not perfectly uniform, SPLs remained within ecologically realistic bounds. We acknowledge, however, that minor SPL variation may have contributed additional variability to the behavioral responses. To minimize the risk of eliciting startle responses from unnaturally close playbacks, we only included trials in which giraffes were positioned at least 30 m from the speaker. At shorter distances, sound reflections and constructive interference can locally amplify acoustic energy, producing levels that exceed those encountered under natural conditions (Marten and Marler 1977). Conversely, attenuation and masking increase sharply beyond about 100 m in vegetated habitats (Yip et al. 2017). Restricting playback distances to 30–100 m therefore ensured that all stimuli were clearly audible while remaining within acoustically and behaviorally realistic bounds.

### 2.5 Playback Experiment Procedure

Playback experiments were conducted under calm and dry weather conditions from dawn to dusk during random times of the day, across all three study sites. Giraffes were selected opportunistically, and only playback trials involving sub‐adults or adults were included in the analysis. There was a minimum of 48‐h between trials at the same study site to avoid habituation due to over exposure. A trial was defined as playing one test stimulus and one control stimulus. To avoid predictability and sampling bias, the test stimulus and the order of the playback were randomly selected using a random number generator.

The playback trials occurred using the same experimental procedure. The giraffe was located from a vehicle then approached on foot. The JBL‐EON compact loudspeaker was placed on a tripod 1 m above the ground and remained out of sight from the giraffes behind dense vegetation (Figure 2). The observer was located parallel between 15 and 55 m from the loudspeaker, and the loudspeaker was located between 30 to 100 m from the giraffe. The giraffe had to be stationary while displaying calm behavior before the stimulus could be played. An Eono PF230 Golf Laser Rangefinder and/or GPS coordinates were used to determine the distance between the giraffe and the loudspeaker. All behavioral observations were recorded using a Sony a6000 camera. The playbacks did not occur in rain or conditions with extreme wind, which was defined as a maximum wind speed of 25 km/h.

One experimental trial was divided into two sections: the first stimulus and the second stimulus. The playback could only start when the giraffe displayed calm behavior, which was noted as feeding or ruminating. Additionally, the observer required a clear view to the focal giraffes, defined as unobstructed visibility of the shoulders, neck, and head, while the observer remained stationary and inconspicuous. Before playing the first stimulus, a 3‐min habituation period was recorded. This habituation period served to verify that the giraffe was in a calm, undisturbed behavioral baseline before playback onset and was therefore not analyzed further. The first stimulus could be played only if the giraffe continued feeding or ruminating after the conclusion of the 3‐min habituation period. After the stimulus ended, the observer recorded a 3‐min recovery period. The observation for the first stimulus was completed, and the second stimulus was completed by following the same procedure.

Individuals rarely remained in the same location after the first stimulus, thus resulting in the observer locating the individuals again and restarting with the habituation process. If the original focal individual could not be located after the first stimulus due to ecological constraints (terrain or dense vegetation), a new focal individual was identified and received the second stimulus. In the case of a second focal individual for the second sound stimulus, that individual was exposed to only one sound on that particular day. In occasional instances of the giraffes not being located after the first stimulus had been played due to ecological constraints, only one stimulus was observed during the trial.

Auditory notes were recorded to document the time of day, the distance between the giraffe and the loudspeaker, group size, and any additional contextual observations. Group size was defined as the number of individuals seen by the observer within a 100 m radius of the focal giraffe individual or group. This 100 m threshold follows Cameron and du Toit (2005), who adopted this distance to define group membership in giraffes. The GPS coordinates recorded the positions of the giraffes, the loudspeaker, and the observer after each playback. External factors, such as people walking or driving and creating distractions for the giraffes, were mitigated as much as possible. However, such interference could not always be avoided, particularly at the FGR site, which included public access areas. If a giraffe looked toward a disturbance for up to 5 s and subsequently reoriented toward the speaker or observer without moving away, the behavior was classified as ‘scanning’ and retained for analysis. In contrast, if the disturbance elicited a stronger reaction, such as looking toward the disturbance for at least 10 s or physically moving away from the area, the trial was terminated and excluded from the dataset.

The study giraffes were given unique identifying codes and identified based on their unique spot patterns. The age of the individuals was estimated using age classes described by Muller et al. (2022): sub‐adults were 12 months to less than 4 years (female *n* = 4, male *n* = 4), adult females were at least 4 years old (*n* = 14), adult males were at least 4 years old (*n* = 14).

### 2.6 Behavioral Analysis

Behavior was coded in BORIS v.7.13.9 (Friard and Gamba 2016) over a 180‐s window beginning at playback onset (120 s of playback + 60 s post‐playback). A detailed ethogram (Table S2) based on behaviors reported in the literature (Seeber et al. 2012) and from field observations was used to identify vigilance‐related responses such as head or neck lifting or turning, orientation toward the sound source, scanning of the surroundings, and locomotor behaviors including approach or displacement movements. These behaviors were established and validated by Baotic and Szipl (2025a, 2025b), whose inter‐observer reliability testing confirmed high scoring consistency.

Each trial was assigned a ‘behavioral reaction index’ on a 0–12 scale. A score of 0 indicated no observable reaction the individual continued feeding or ruminating, whereas 12 denoted the most intense response, typically involving abrupt interruption of natural behavior and movement away from the stimulus. For descriptive clarity, scores were grouped into three biologically meaningful categories (A = 0–5, B = 6–9, and C = 10–12). Category A reflects no reaction or minimal behavioral change, with continued natural activity and at most brief attention toward the stimulus; Category B indicates cessation of natural behavior and sustained attention toward the stimulus; and Category C represents a full‐vigilance response including relocation away from the stimulus. A tabular overview of the reaction‐intensity scoring categories is provided in Table 3.

**TABLE 3:** Behavioral reaction scoring system used to quantify giraffe responses to playback stimuli. Scores ranged from 0 to 12 and were grouped into three intensity levels. Low‐level responses (0–5) reflected minimal vigilance or no behavioral change. Moderate responses (6–9) indicated clear vigilance or investigatory behavior directed toward the stimulus. High‐intensity responses (10–12) represented strong avoidance reactions, including rapid withdrawal or flight. Operational definitions and anchor examples are provided for each score.

Score category	Operational definition and anchor examples
0–5	0: no reaction, continues feeding/ruminating also includes drinking, lying, necking, osteophagia, scratching, ‘head to observer while ruminating’, ‘out of sight at beginning/following feeding’, scanning at the beginning of the playback 1: one ear moves 2: both ears move 3: walks toward trees to continue browsing 4: scanning the area not related to the stimulus (isolated scanning) 5: turns head while still chewing/ruminating (to speaker/observer/other giraffes)
6–9	6: stops chewing/ruminating and looks (head to observer/speaker/other giraffes) 7: turns body posture (body to speaker/other giraffes/observer, includes standing) 8: walks closer, begins to investigate (toward speaker/observer, may be while ruminating) 9: moves off gradually (walks away from speaker for < 15 s, or walks toward other giraffes while ruminating)
10–12	10: instant move out of sight/hearing (walks away from speaker for ≥ 15 s or walk away immediately followed by out of sight) 11: quick move toward other animals (safety in numbers) 12: run away (rapid retreat/flight)

To quantify temporal aspects of the responses, video annotations were exported as one–zero samples at 1‐s resolution across the 180‐s window (Altmann 1974). Latency to first response was defined as the first occurrence of any target behavior after playback onset. Because large herbivores typically show rapid responses to novel or potentially threatening stimuli (Palmer and Packer 2021), we focused on the initial part of the trial to capture the immediate vigilance response.

Beginning at playback onset, we identified the first three distinct behaviors that met our reaction criteria and recorded for each the reaction intensity (0–12 score) and its latency in seconds. To emphasize acute, stimulus‐evoked responses, we weighted intensities by a decreasing logistic function centered at 12 s, giving full weight to reactions within ~9 s and progressively less weight to those occurring later, with minimal weight after ~15 s. For example, a head‐up at 3 s received full weight, whereas the same behavior at 20 s received zero weight and was not considered a direct reaction to the stimulus. The highest resulting time‐weighted reaction score served as the primary response variable. The exact function is provided in the Supporting Information: Equation S1.

This analytical approach captures the magnitude, timing, and immediacy of vigilance responses while minimizing the influence of delayed or residual behaviors.

### 2.7 Statistical Analysis

#### 2.7.1 Data Processing and Feature Construction

Statistical analyses were conducted in R software (R Core Team 2024) using the ‘rstanarm’ package (Goodrich et al. 2025). Behavioral response data were derived from the binary‐coded video observations (see source data). From this raw data, a set of ‘engineered variables’ (features) was created to quantify behavioral latency, duration, and reaction intensity. These features are a numerical input acting as a bridge between the raw data and the model (Zheng and Casari 2018). In other words, these engineered variables represent the three quantitative response measures extracted from the behavioral time series: latency to first response, reaction duration, and time‐weighted reaction intensity. These features served as dependent variables in subsequent linear mixed models. Reaction duration (in seconds) was measured directly from video annotations and modeled as a continuous variable without truncation or censoring. Duration values were highly variable across individuals and trials, resulting in wide 95% credible intervals (CrI) and no consistent differences between stimuli or locations. Because reaction duration did not provide a reliable or interpretable pattern see Tables S3, S4, the main analyses focus on reaction intensity, which showed clearer and more robust effects of stimulus type. Full duration results are presented in the Supporting Information for transparency.

#### 2.7.2 Raw‐Model Structure

To assess whether acoustic and contextual factors predicted giraffe behavioral responses, we fitted Bayesian mixed‐effects linear models. To account for potential pseudoreplication, the models incorporated an additive random‐effects structure including ‘Giraffe ID’, ‘Playback event ID’ (video number), and ‘Playback exemplar ID’ (the eight unique sound files per stimulus type). ‘Giraffe ID’ captured repeated measures of the same individual across different playbacks, ‘Playback event ID’ controlled for shared contextual conditions when multiple giraffes were recorded responding within the same playback event, and ‘Playback exemplar ID’ accounted for variation among the sound files that were reused across different trials. These effects were not nested, as individuals, playback events, and exemplars intersected across observations.

All playbacks were conducted with giraffes positioned between 30 and 100 m from the speaker. Within this range, sound levels represent typical field conditions and are audible but non‐startling to giraffes. Fixed effects included sound type (categorical), study location, group size, wind speed, sex‐age class, speaker distance, and date. Date (standardized within location and sound type) was included to account for systematic temporal variation across the data‐collection period.

#### 2.7.3 Covariates: Standardization

A key interaction between sound type and location was included to test whether responses varied depending on exposure history, since different locations may reflect different levels of habituation to certain anthropogenic sounds. Continuous predictor variables (group size, speaker distance, and wind speed) were standardized to improve model convergence and make effect sizes comparable across predictors by subtracting the mean and dividing by the standard deviation (mean = 0, SD = 1). Because sound attenuation with distance is non‐linear, we allowed distance to have a curved effect by modeling it with a second‐degree polynomial term. Sex and age were combined into a biologically relevant categorical variable with four levels: adult male, adult female, subadult male, and subadult female. Individuals were assigned to these categories based on body size, ossicone morphology, and pelage characteristics observed in the field (Muller et al. 2022).

#### 2.7.4 Modeling Framework and Pairwise Contrasts

All models were fitted using Bayesian estimation with standard non‐informative or weakly informative priors as automatically implemented by the software. Markov Chain Monte Carlo (MCMC) diagnostics confirmed model stability, with Rhat values ranging from 1.000 to 1.001 and Monte Carlo standard errors (MCSE) below 0.023 (Flegal et al. 2008).

Pairwise contrasts were computed to investigate differences between stimulus types, locations, and possible effects of sex and age (Flegal et al. 2008). The contrasts were calculated within location and averaged over sex‐age categories while holding control variables (e.g., wind speed, speaker distance, and date) constant at their mean values to facilitate model interpretability and ensure comparability across conditions. All within‐location pairwise contrasts were derived from the full sound type × location interaction model and do not represent separate analyses conducted independently for each site. Because all models were fitted within a Bayesian framework, we report posterior means (*β*) and 95% CrI for all fixed effects and contrasts. Statistical support for an effect was inferred when the CrI did not include zero. For contrasts, we report the posterior expected difference between conditions, denoted *μ*_diff_. This value represents the posterior mean of the contrast distribution and therefore reflects a model‐predicted difference rather than a fixed‐effect coefficient. Accordingly, *β* notation is reserved for regression coefficients, and *μ*_diff_ is used exclusively for contrast estimates. We did not report *p*‐values, as they do not have a meaningful interpretation under Bayesian inference and can provide misleading evidence when applied to posterior distributions (Marsman and Wagenmakers 2017). Instead, CrI provide a direct probability statement about the parameter values and therefore offer an appropriate and transparent measure of uncertainty for Bayesian models. Pairwise contrasts were summarized using their posterior means and corresponding credible intervals.

#### 2.7.5 Matched‐Model Analysis (Mahalanobis Distance Matching)

To evaluate whether giraffes responded more strongly to anthropogenic sounds than to the natural control stimulus, we conducted a pseudo‐controlled comparison using ‘Mahalanobis distance matching’, an approach increasingly used in ecological and behavioral field studies where full randomization is not feasible (e.g., Etherington 2021). Dove control trials were unevenly available across sites and sampling periods, and anthropogenic and control stimuli were not always presented under comparable environmental conditions. Matching was therefore used to obtain controlled, comparable contrasts between sound types by pairing observations that are closest in multivariate covariate space while accounting for correlations among covariates (Amusa et al. 2022). Although the experimental design aimed to present each individual with both a test stimulus (vehicle, drone, or human voice) and a control stimulus (dove call) under similar conditions, logistical constraints made this inconsistent. To address this, we used the Mahalanobis distance to match each anthropogenic test trial to the most similar available dove trial from the same individual. Matching was based on wind speed, speaker distance, group size, sex‐age class, and date (which weighted most heavily to prioritize temporally close trials). This procedure allowed us to reduce confounding by aligning the environmental context of test and control stimuli, thereby approximating a controlled within‐subject comparison. For each matched pair, we subtracted the reaction score of the dove trial from the anthropogenic trial, yielding a difference score that reflects the relative strength of giraffe response to anthropogenic versus natural sounds.

Following the construction of matched test‐control pairs, we visualized modeled effects using partial‐dependence predictions. Continuous contextual covariates (group size, wind speed, speaker‐giraffe distance, and date) were held at their sample means, yielding fitted values that reflect the expected response of an average giraffe, marginalized over sex and age class, under typical environmental conditions. Location, sound type, and sex‐age class were the primary predictors of interest, with remaining covariates fixed to aid interpretability. Observed values were superimposed to show correspondence between the data and the fitted effects.

## 3 Results

A total of 33 individual giraffes were observed across the three study sites (APGR: 19 individuals, FGR: 4, WGL: 10). Across all trials, 226 videos were obtained, comprising 477 individual giraffe observations included in the final analysis. Site‐level summaries of video trials and stimulus‐specific individual counts are provided in Table 4.

**TABLE 4:** Number of video recordings and giraffe individuals observed for each sound stimulus at each study site. Each cell reports the number of video trials followed by the number of individuals contributing to that stimulus category (videos|individuals). Because the same giraffes were exposed to multiple stimulus types, the summed stimulus‐level individual counts exceed the true number of unique individuals at each site.

Location	Drone	Vehicle	People talking	Ring‐neck dove	Total videos	Sum of stimulus‐level individuals	Unique individuals
APGR	6|7	5|4	5|8	15|15	31	34	19
FGR	14|4	19|4	18|4	53|4	104	16	4
WGL	19|10	18|10	19|10	57|10	113	40	10

*Note:* ‘Individuals’ reflects the number of different giraffes contributing to each stimulus category. Because most individuals experienced multiple stimulus types, the summed stimulus‐level totals exceed the true number of unique giraffes per site. The rightmost column reports the actual number of unique giraffes at each reserve.

### 3.1 Models Based on the Raw Observations

Across the full dataset, there was no evidence for a consistent effect of individual identity on behavioral responses, and several contextual variables also showed no significant influence. Group size (*β* = −0.171, 95% CrI: −0.488 to 0.158) and wind speed (*β* = 0.054, 95% CrI: −0.287 to 0.191) had credible intervals that overlapped zero. In contrast, speaker distance showed clear posterior support for an effect on reaction intensity, with giraffes responding more strongly when the sound source was closer. All contextual variables were retained in the model to control for potential confounding effects.

### 3.2 Reaction Duration

Reaction duration was highly variable across trials and did not exhibit a consistent stimulus‐dependent structure. Detailed model summaries of duration are presented in the Tables S3, S4. Consistently across the raw (Table S3) and the matched‐model analyses (Table S4), none of the anthropogenic stimuli produced supported increases in reaction duration relative to the dove control, with all 95% CrIs overlapping zero. Thus, in contrast to reaction intensity, duration did not yield statistically supported fixed effects of sound type. Together, these results indicate substantial variability in duration, reinforcing reaction intensity as the more reliable behavioral metric for evaluating giraffe responses to anthropogenic sound.

Nevertheless, descriptive raw durations revealed site‐dependent trends that broadly mirrored the intensity patterns. At APGR, anthropogenic stimuli elicited the longest vigilance responses (drone: mean 46.6 ± 32.9 s, *n* = 11; talking: 26.7 ± 29.8 s, *n* = 10; vehicle: 13.3 ± 10.1 s, *n* = 7), whereas dove calls produced very short responses (0.5 ± 1.4 s, *n* = 31). Duration responses at FGR were shorter and more variable (drone: 21.2 ± 23.7 s, *n* = 22; talking: 15.6 ± 32.7 s, *n* = 35; vehicle: 9.4 ± 16.0 s, *n* = 32; dove: 4.1 ± 9.4 s, *n* = 85). At WGL, reactions were generally brief across all stimuli (drone: 12.3 ± 20.7 s, *n* = 41; talking: 7.2 ± 9.0 s, *n* = 42; vehicle: 8.1 ± 14.5 s, *n* = 37; dove: 4.6 ± 13.2 s, *n* = 123).

### 3.3 Reaction Intensity

Predicted reaction intensities from the raw model showed that giraffes generally reacted more strongly to anthropogenic sounds (drone, vehicle, and human voices) than to the natural control (dove coo calls). A plot of raw reaction intensities across age‐sex classes and sites is provided in Figure S1. However, the strength of these effects varied markedly across sites. These differences among locations arise from the sound type × location interaction included in the full Bayesian model. Within‐site pairwise contrasts revealed that giraffes at APGR responded most strongly to anthropogenic sounds, with all three stimuli eliciting clear increases in reaction intensity relative to dove calls (drone: *μ*_diff_ = 4.266, CrI: 2.679–5.803; vehicle: *μ*_diff_ = 5.294, CrI: 3.495–7.084; talking: *μ*_diff_ = 1.884, CrI: 0.281–3.505). At WGL, responses were positive but generally weaker, with drone and talking stimuli producing moderate increases relative to dove calls (drone: *μ*_diff_ = 1.122, CrI: −0.284–2.018; talking: *μ*_diff_ = 1.326, CrI: 0.436–2.192), while vehicle responses were smaller and more uncertain (*μ*_diff_ = 0.948, CrI: 0.062–1.884). At FGR, anthropogenic sounds produced small but mostly positive effects (drone: *μ*_diff_ = 2.088, CrI: 0.970–3.199; talking: *μ*_diff_ = 1.178, CrI: 0.190–2.125; vehicle: *μ*_diff_ = 1.065, CrI: −0.108–2.082), although the associated uncertainty was greater than at the other sites. Additional within‐site contrasts among anthropogenic sound types (e.g., drone vs. talking, vehicle vs. drone) are consistent with this pattern, indicating generally higher responsiveness to drone and vehicle noise at APGR and weaker differentiation among stimuli at WGL and FGR. Complete within‐site pairwise contrasts for the raw reaction‐intensity model are provided in Table S5. Between‐site comparisons showed that overall reaction intensities were highest at APGR, moderate at WGL, and lowest at FGR, consistent with differences in ecological exposure history (Table S6).

Taken together, the raw reaction‐intensity model revealed a clear gradient in responsiveness across sites and stimulus types, with the strongest anthropogenic effects occurring at APGR and much weaker responses at WGL and FGR. To provide a controlled comparison between anthropogenic sounds and dove calls within each site, we additionally analyzed reaction differences using a matched‐design model.

### 3.4 Matched‐Model Analysis of Reaction Intensity

**FIGURE 3:**
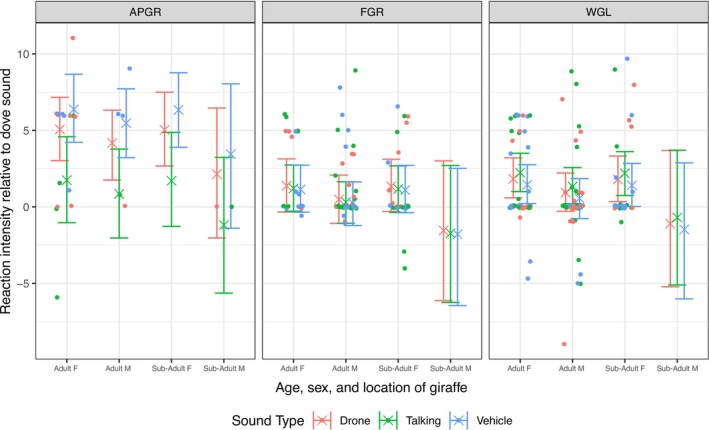
Matched comparison of reaction intensity relative to the control stimulus. Reaction‐intensity differences between anthropogenic stimuli (drone, vehicle, talking) and the matched dove control are shown across age‐sex classes for three study sites (APGR, FGR, WGL). Values reflect model predictions derived from Mahalanobis‐matched trials from the same individuals, controlling for group size, speaker distance, and wind speed. Positive values indicate stronger reactions to anthropogenic stimuli.

The matched‐model analysis estimates how each anthropogenic sound affects reaction intensity relative to matched dove control trials within each site. Figure 3 illustrates the matched reaction‐intensity differences for each anthropogenic stimulus across age‐sex classes and study sites relative to the control stimulus. Model predictions from the matched‐design analysis showed the same site‐dependent pattern observed in the raw models. At APGR, both drone and vehicle noise produced clear increases in reaction intensity relative to the matched dove controls (drone: *β* = 4.104, 95% CrI: 1.354–6.861; vehicle: *β* = 5.406, CrI: 2.482–8.299), while the effect of people talking remained uncertain (*β* = 0.778, CrI: −2.495 to 4.117). At WGL, all anthropogenic stimuli resulted in positive mean differences compared to controls, but the 95% CrIs for drone (*β* = 0.866, CrI: −1.144 to 3.106), talking (*β* = 1.268, CrI: −0.786 to 3.348), and vehicle noise (*β* = 0.483, CrI: −1.635 to 2.580) all overlapped zero, indicating weak or no posterior support for clear effects. At FGR, none of the anthropogenic stimuli elicited supported increases relative to the controls, with all CrIs crossing zero (drone: *β* = 0.417, CrI: −1.961 to 2.833; talking: *β* = 0.238, CrI: −1.991 to 2.441; vehicle: *β* = 0.172, CrI: −2.099 to 2.396). Sex‐age class had no supported effect on reaction intensity in either the raw or matched models (all 95% CrIs overlapped zero, Table S7). Because these demographic effects were weak and non‐directional, we report them only in the Supporting Information. Further, overall, the matched‐model contrasts corroborated the raw‐model results, confirming strong responses at APGR, weaker and uncertain responses at WGL, and minimal responses at FGR.

### 3.5 Additional Contrasts Among Anthropogenic Stimuli and Between Sites

To further characterize stimulus‐specific and population‐level patterns, we examined secondary contrasts among anthropogenic sounds within sites and across sites.

Within APGR, giraffes also discriminated among anthropogenic sounds: responses to vehicle and drone noise were stronger than responses to people talking (vehicle–talking: *μ*_diff_ = 4.628, CrI: 1.180–8.065; drone–talking: *μ*_diff_ = 3.326, CrI: 0.064–6.413), whereas differences between vehicle and drone responses were small and uncertain (vehicle–drone: *μ*_diff_ = 1.302, CrI: −1.580–4.289). Within WGL and FGR, no supported differences among anthropogenic stimuli were detected, reflecting the generally low and relatively uniform responsiveness at these more human‐exposed sites (Table S8).

Between‐site contrasts revealed a consistent population‐level gradient in sensitivity. For both vehicle and drone noise, giraffes at APGR reacted substantially more strongly than those at WGL or FGR (e.g., vehicle: APGR–FGR *μ*_diff_ = 5.234, CrI: 2.704–7.773, APGR–WGL *μ*_diff_ = 4.923, CrI: 2.574–7.356; drone: APGR–WGL *μ*_diff_ = 3.238, CrI: 0.877–5.478, APGR–FGR *μ*_diff_ = 3.688, CrI: 0.958–6.414), whereas differences between WGL and FGR were small and uncertain (Table S9).

Together, these secondary contrasts indicate that both the magnitude of anthropogenic noise responses and the ability to discriminate among noise types are greatest at APGR and decline with increasing exposure history, reinforcing the habituation gradient inferred from the primary anthropogenic‐dove comparisons.

## 4 Discussion

This study provides experimental evidence that anthropogenic noise alters vigilance behavior in free‐roaming giraffes and that the strength of these responses varies with both the type of sound and the ecological exposure history of the population. Across all sites, giraffes responded more strongly to anthropogenic than to natural control sounds, demonstrating that acoustic cues alone, without visual stimuli, are sufficient to interrupt ongoing behavior. The magnitude of these responses depended on local disturbance levels, with giraffes at the low‐exposure site showing the strongest and most differentiated reactions, and individuals at more human‐exposed sites showing weaker and less distinct responses among sound types. These patterns are consistent with work showing that antipredator strategies and vigilance intensity vary with predator traits and perceived risk, and embedded within dynamic landscapes of fear shaped by space, time and ecological context (Frid and Dill 2002; Blumstein 2006; Palmer and Packer 2021; Palmer et al. 2022). More specifically, they reinforce the view that acoustic signal properties and ecological experience jointly shape how large herbivores perceive and evaluate acoustic risk (Blumstein and Récapet 2009; Shannon et al. 2016; Baotic and Szipl 2025a, 2025b). Although we did not explicitly model acoustic structure, the observed differences among stimuli were consistent with expectations based on their spectral and temporal properties (Morton 1977; Francis and Barber 2013). Mechanical noise from drones and vehicles elicited the highest vigilance, human voices produced moderate responses, and dove calls consistently evoked the weakest reactions. These patterns highlight that both familiarity and the ecological relevance of specific sound types contribute to perceived risk.

### 4.1 Site‐ and Stimulus‐Specific Responsiveness

Vigilance responses exhibited a clear gradient across the three study sites. At APGR, the most remote site with minimal daily human presence, giraffes showed the strongest and most reliable increases in vigilance to all anthropogenic sounds. This heightened sensitivity aligns with patterns observed in low‐disturbance environments, where novel or unpredictable stimuli elicit stronger anti‐predator responses due to limited opportunities for habituation (Frid and Dill 2002; Brown et al. 2012; Shannon et al. 2016). At APGR, anthropogenic noise represents an atypical cue in a largely natural soundscape and is therefore likely to be interpreted as a potential threat.

At WGL, giraffes experience constant highway noise and relatively limited direct human presence. Their responses were weaker and less differentiated than at APGR, but still elevated relative to the dove control. Notably, vehicle noise elicited stronger vigilance than might be expected from continuous background exposure. This suggests that habituation to distant, low‐intensity traffic does not necessarily generalize to sudden or nearby mechanical sounds (Brown et al. 2012; Francis and Barber 2013; Martin et al. 2022).

Giraffes at FGR, the most urbanized site, showed the weakest vigilance responses, consistent with broad habituation to frequent human activity. However, drone noise elicited comparatively stronger responses than the other anthropogenic sounds at FGR, although the overall effect remained small in the matched model. This pattern mirrors findings from other species showing that UAVs can provoke behavioral responses in both naïve and human‐habituated populations (Bennitt et al. 2019; Mesquita et al. 2022; vanVuuren et al. 2023; Stepien et al. 2024; Afridi et al. 2025). This heightened salience is likely driven by the broadband and mechanically generated acoustic structure of drone noise, which differs markedly from natural ambient sound and lacks the stable harmonic features typical of biological calls (Duporge et al. 2021). Its ecological novelty and atypical spectral profile may further amplify these responses across taxa (Afridi et al. 2025).

Beyond site‐level differences, stimulus‐specific patterns further highlight how sound properties interact with ecological context. Drone and vehicle noise elicited the strongest vigilance, particularly at APGR, which is consistent with the fact that broadband, noisy mechanical sounds function as ambiguous cues that can signal the movement or presence of large animals or potential threats (Morton 1977; Halfwerk et al. 2011; Francis and Barber 2013). This is consistent with broader evidence that spectrally complex or noisy signals evoke heightened arousal across vertebrates, helping explain the salience of mechanical noise in this context (Massenet et al. 2025). Human voices produced moderate responses across sites, matching evidence that mammals often treat human vocalizations as indicators of danger (Zanette and Clinchy 2019; Reilly et al. 2022; Zanette et al. 2023). Dove calls, by contrast, evoked minimal vigilance, confirming their suitability as a familiar and benign natural control sound (Slabbekoorn et al. 1999; Shieh et al. 2016).

Because individuals sometimes responded in close proximity, some degree of shared behavioral influence is inevitable in naturalistic field settings. Our analytical approach accounts for these contextual effects, but residual dependence is a normal feature of field data and is interpreted conservatively here.

Because individuals sometimes responded in close proximity, some degree of shared behavioral influence is inevitable in naturalistic field settings (Bejder et al. 1998; Farine and Whitehead 2015). Our modeling approach accounts for these contextual effects, and any remaining dependence is typical of free‐ranging behavioral data and is unlikely to change the overall interpretation of the results.

Overall, these patterns demonstrate that giraffes integrate ecological experience, cue familiarity, and sound‐type characteristics when assessing acoustic risk. This interpretation aligns with recent experimental evidence showing that giraffe responses to biologically relevant acoustic cues, including oxpecker alarm calls and lion roars, are similarly shaped by population‐specific predator exposure and learned associations (Baotic and Szipl 2025a, 2025b). Across these studies, giraffes from low‐disturbance or predator‐experienced populations show stronger and more differentiated vigilance responses than individuals from human‐dominated or predator‐free environments. The present findings extend this pattern to anthropogenic noise, indicating that the same experience‐based mechanisms that structure responses to natural risk cues also modulate how giraffes perceive and evaluate human‐generated sounds.

Beyond these mechanistic insights, increased vigilance, even when brief, reduces time available for foraging and social activities, with potential consequences for space use and social dynamics (Muller et al. 2019; Bond et al. 2023; Brown et al. 2023). In environments with higher human presence, habituation reduces responsiveness to common anthropogenic sounds, but this habituation does not uniformly extend to all sound types. Understanding these context‐ and stimulus‐dependent patterns is therefore critical for anticipating how giraffes respond to expanding acoustic disturbance.

### 4.2 Conservation Implications and Future Directions

The behavioral changes observed in this study highlight the importance of recognizing anthropogenic noise as a relevant ecological stressor in giraffe habitats. Elevated vigilance, although adaptive in contexts of perceived risk, comes at the expense of foraging, social interaction, and calf guarding, creating potential opportunity costs that may accumulate in chronically disturbed environments (Lima and Dill 1990; Olson et al. 2015; Freidly et al. 2025). In low‐disturbance areas, where unfamiliar or inconsistent cues remain particularly salient, even brief vigilance responses may alter local space use or movement decisions, similar to noise‐driven behavioral shifts documented in other large herbivores (Ajibola‐James et al. 2024; Zeller et al. 2024). These considerations are especially relevant for giraffe populations inhabiting small or fragmented reserves, where limited space for movement can intensify the effects of disturbance.

Accordingly, soundscape management therefore represents a practical tool for mitigating human impacts. Although protected areas offer some buffering from human activity, many reserves experience increasing noise intrusion from roads, tourism, and adjacent development (Hatch and Fristrup 2009; Buxton et al. 2019; Rice et al. 2020). Maintaining low‐noise zones around key foraging areas, travel corridors, and nursery sites may reduce disturbance, while vegetated barriers and strategic placement of infrastructure could help preserve acoustic refuges (Francis and Barber 2013; Kunc and Schmidt 2019; Ajibola‐James et al. 2024). Visitor management, particularly limiting engine idling, loud talking, or off‐road vehicle use, may further reduce acute disturbances in sensitive areas.

Drone use warrants special consideration, particularly in areas with sensitive wildlife populations. UAVs are increasingly deployed for wildlife monitoring, anti‐poaching, and tourism (Mo and Bonatakis 2021), yet growing evidence, including the present study, shows that drone noise can disturb ungulates regardless of prior human exposure (Mesquita et al. 2022; vanVuuren et al. 2023; Afridi et al. 2025). Implementing conservative operational guidelines, such as maintaining higher flight altitudes, lateral offsets from individuals and groups, avoiding hovering, and selecting quieter platforms, will help minimize disturbance (Duporge et al. 2021). Particular caution is warranted in low‐disturbance reserves, where drone noise may be especially salient.

Future work should aim to quantify the physiological and longer‐term consequences of noise exposure for giraffes. Although giraffes show elevated vigilance and stress‐related endocrine changes in response to human presence (Scheijen et al. 2021), the specific contribution of noise remains unclear. In other taxa, anthropogenic noise can elevate glucocorticoids, impair cognitive performance, alter movement and foraging behavior, and reduce reproductive success (Gill et al. 2014; Shannon et al. 2016; Arcangeli et al. 2023). Longitudinal approaches integrating behavioral, hormonal, spatial, and acoustic monitoring will be essential for assessing the cumulative impacts of noise and informing soundscape‐based management strategies in increasingly human‐modified savanna ecosystems.

## 5 Conclusion

Our multisite playback experiment reveals that anthropogenic noise is a potent behavioral disruptor for free‐roaming giraffes and that responsiveness to noise is fundamentally shaped by ecological exposure history. Giraffes inhabiting low‐disturbance environments mounted the strongest vigilance responses to mechanical and human‐generated sounds, demonstrating that experience and familiarity strongly influence how large herbivores evaluate acoustic risk. These findings show that noise sensitivity is not fixed but emerges from the interaction between environmental context and the acoustic characteristics of disturbance cues. Although giraffes served here as a model species, the mechanisms identified, including experience‐dependent vigilance, stimulus‐specific risk assessment and limited opportunities for habituation, are shared across many taxa that rely on acoustic information to detect threats. As human‐generated noise expands across savanna ecosystems, integrating soundscape considerations into wildlife management, reserve planning, tourism practices and UAV operations will be essential. Future research should link behavioral responses to physiological stress, movement patterns and demographic outcomes to clarify how noise influences survival, reproduction and population persistence, and to support effective conservation planning.

## Author Contributions

**Kaitlyn Taylor:** data curation (lead), investigation (lead), methodology (supporting), project administration (lead), writing – original draft (lead). **Sean van der Merwe:** formal analysis (lead), methodology (supporting), software (lead), validation (lead), visualization (equal), writing – review and editing (supporting). **Andri Grobbelaar:** investigation (supporting), resources (supporting), supervision (supporting), writing – original draft (supporting). **Francois Deacon:** conceptualization (supporting), investigation (supporting), methodology (supporting), resources (lead), supervision (equal), writing – original draft (supporting). **Anton Baotic:** conceptualization (lead), data curation (supporting), investigation (supporting), methodology (lead), resources (lead), software (supporting), supervision (equal), visualization (supporting), writing – original draft (supporting), writing – review and editing (lead).

## Funding

This research was funded in whole or in part by the Austrian Science Fund (FWF) (grant‐doi 10.55776/P36120). For open access purposes, the author has applied a CC BY public copyright license to any author‐accepted manuscript version arising from this submission.

## Ethics Statement

This study was approved by the Animal Research Ethics Committee of the University of the Free State, South Africa (reference numbers: UFS‐AED2022/0045/23).

## Conflicts of Interest

The authors declare no conflicts of interest.

## Supporting information


**Appendix S1:** ece372763‐sup‐0001‐AppendixS1.zip.

## Data Availability

Source data including behavioral datasets and analysis scripts are provided in the Supporting Information in open formats to allow full reproducibility.
