# Supplementary material for: Ecological Exposure History Shapes Giraffe Vigilance Responses to Anthropogenic Noise: A Multisite Playback Experiment
Source: Ecol Evol. 2025 Dec 17;15(12):e72763. doi: 10.1002/ece3.72763 (PMC12711595; doi:10.1002/ece3.72763)

Supplementary Material

Ecological Exposure History Shapes Giraffe Vigilance Responses to Anthropogenic Noise: A Multisite Playback Experiment

Tables S1-S9:

Table S1. Exemplar usage. Number of playback trials per exemplar across the four stimulus categories (dove calls, human voices, vehicle noise, and drone noise). All exemplars were randomly rotated across trials; variation in usage reflects differences in animal availability and trial feasibility.

Category	Exemplar	Uses
Dove	Dove1	17
Dove	Dove2	19
Dove	Dove3	41
Dove	Dove4	28
Dove	Dove5	41
Dove	Dove6	30
Dove	Dove7	33
Dove	Dove8	30
Drone	Drone1	17
Drone	Drone2	2
Drone	Drone3	9
Drone	Drone4	9
Drone	Drone5	17
Drone	Drone6	6
Drone	Drone7	5
Drone	Drone8	9
Talking	Talking1	7
Talking	Talking2	17
Talking	Talking3	19
Talking	Talking4	10
Talking	Talking5	9
Talking	Talking6	6
Talking	Talking7	7
Talking	Talking8	12
Vehicle	Vehicle1	16
Vehicle	Vehicle2	11
Vehicle	Vehicle3	9
Vehicle	Vehicle4	9
Vehicle	Vehicle5	7
Vehicle	Vehicle6	10
Vehicle	Vehicle7	8
Vehicle	Vehicle8	6

Table S2. Ethogram of behaviours used in the video analysis. All behaviours are state event.

Behavioral category	Behavior code	Description
Body movement	Body turns towards speaker	Animal repositions itself to turn the body towards the speaker direction
	Body turns to other giraffes	Animal repositions itself so its body is facing other giraffes
	Body turns to observer	Animal repositions itself to face the observer
Calm Behaviour	Chewing/ruminating	Animal chews or ruminates
	Feeding	Animal continues to browse
	Drinking	Animal drinks water
Ear movement	Two ears move	Two ears move towards the noise
	One ear moves	One ear moves towards the noise
Feeding Levels	Level 1 feeding	Feeding from the ground
	Level 2 feeding	Feeding from knee height
	Level 3 feeding	Feeding from chest height
	Level 4 feeding	Feeding from neck height
	Level 5 feeding	Feeding from head height
	Level 6 feeding	Feeding from stretch height, head reaching upwards
Head movement	Head moves toward speaker	Head moves to look at speaker
	Head moves towards another giraffe	Head turns to look at other giraffes
	Head moves to observer	Head turns to look at the observer
	Head to speaker while ruminating	The head turns towards the speaker while giraffe is ruminating
	Head to observer while ruminating	The head turns towards the observer while ruminating
	Head moves towards other giraffes while ruminating	The head moves to look to the other giraffes while ruminating
	Head 45	Head is positioned 45 degrees
	Head 90	Head is positioned 90 degrees
	Head 180	Head is positioned 180 degrees
	Head 135	Head is positioned 135 degrees
Neck Position	Neck in S position	Animal pulls head back into a defensive "S" shape
	Neck low 45	Neck is positioned at 45 degrees
	Neck medium 90	Neck is positioned 90 degrees
	Neck 135	Neck is positioned at 135 degrees
	Neck high 180	Neck is 180 degrees with the head extended up
Out of sight	Out of sight	Animal is out of sight
Run	Run away	Animal runs away
Self-grooming	Scratching itself	Individual is brushing against vegetation and/or scratching any part of itself
Standing/Laying	Standing	Animal stands, only after it has been laying down
	Laying down	Animal is laying down
Vigilant	Scanning	Animal is vigilant and scanning the area
Walking	Walks towards speaker	Animal approaches the speaker
	Walks to other giraffes	Animal approaches nearby giraffes
	Walks away from the speaker	Animal walks away from the speaker
	Walks towards observer	Animal approaches observer
	Walk towards speaker while ruminating	The giraffe is ruminating while walking towards the speaker
	Walk away from speaker while ruminating	The giraffe walks away from speaker while ruminating
	Walk towards observer while ruminating	The giraffe is ruminating while walking towards the observer
	Walks towards other giraffes while ruminating	Animal approaches nearby giraffes while ruminating
	Walk towards trees to browse	Animal is walking towards trees to browse
	Walks to trees to browse while ruminating	Animal moves to trees to browse while ruminating

Table S3. Raw-model posterior estimates for reaction duration (seconds). Posterior means (β) and 95% credible intervals (CrI) from the Bayesian mixed-effects model predicting absolute reaction duration from the full dataset. Positive coefficients indicate longer durations relative to the model reference level (APGR, Dove, adult female, mean covariates). Random-effects estimates (Giraffe ID, Playback event, Playback exemplar, and residual variance) are included to characterise variation across individuals and trials. CrIs for all sound-type coefficients overlap zero, indicating no supported effects of anthropogenic stimuli on reaction duration in the raw data.

Term	Mean (β)	Lower 95% CrI	Upper 95% CrI	*P*	Median	Mode	SE	MAD
(Intercept)	3.805	-5.707	13.004	0.418	3.845	4.393	4.757	3.162
LocationFGR	0.349	-10.09	11.236	0.951	0.327	-0.179	5.45	3.647
LocationWGL	1.677	-7.301	11.106	0.719	1.67	1.338	4.771	3.194
Sound_TypeDrone	38.596	22.248	53.91	0	38.566	38.386	8.134	5.492
Sound_TypeTalking	31.013	13.643	47.804	0.002	31.19	32.722	8.874	5.831
Sound_TypeVehicle	11.2	-5.029	29.042	0.2	11.093	10.872	8.859	6.045
Std_Date	1.879	-6.138	9.487	0.635	1.916	2.514	4.043	2.771
Sex_AgeAdult_M	-0.532	-4.038	3.223	0.775	-0.497	-0.38	1.846	1.232
Sex_AgeSubAdult_F	-1.827	-6.299	2.54	0.408	-1.845	-1.722	2.251	1.483
Sex_AgeSubAdult_M	0.967	-11.76	14.426	0.893	0.877	-0.028	6.706	4.434
Std_grp_sz	-2.283	-5.416	0.892	0.16	-2.288	-2.378	1.635	1.091
d_poly_speaker_dist1	-12.24	-62.08	40.395	0.639	-12.264	-13.051	26.03	17.67
d_poly_speaker_dist2	23.96	-22.83	70.358	0.316	24.086	26.629	23.77	15.98
Std_Wind_speed	0.688	-1.823	3.223	0.588	0.706	0.854	1.281	0.856
LocationFGR:Sound_TypeDrone	-20.814	-39.69	-4.706	0.024	-20.75	-20.582	8.936	5.981
LocationWGL:Sound_TypeDrone	-28.032	-44.58	-10.852	0.002	-28.084	-29.084	8.687	5.885
LocationFGR:Sound_TypeTalking	-18.988	-37.68	-1.119	0.044	-19	-18.502	9.39	6.21
LocationWGL:Sound_TypeTalking	-27.113	-44.92	-8.706	0.006	-27.237	-28.922	9.316	6.24
LocationFGR:Sound_TypeVehicle	-5.156	-24.39	12.769	0.595	-5.178	-5.322	9.551	6.461
LocationWGL:Sound_TypeVehicle	-5.38	-24.55	12.614	0.573	-5.36	-4.941	9.513	6.433
LocationFGR:Std_Date	-2.675	-12.11	5.482	0.556	-2.736	-3.583	4.523	3.121
LocationWGL:Std_Date	0.303	-8.336	9.014	0.957	0.226	0.048	4.451	3.041
Sound_TypeDrone:Std_Date	-19.702	-34.46	-4.896	0.01	-19.808	-20.318	7.512	5.045
Sound_TypeTalking:Std_Date	8.249	-7.34	22.81	0.293	8.244	9.045	7.776	5.322
Sound_TypeVehicle:Std_Date	3.315	-14.46	22.929	0.715	3.329	2.649	9.534	6.327
LocationFGR:Sound_TypeDrone:Std_Date	28.18	11.482	45.43	0.001	28.205	27.921	8.64	5.804
LocationWGL:Sound_TypeDrone:Std_Date	17.131	0.837	33.63	0.041	17.194	17.317	8.395	5.665
LocationFGR:Sound_TypeTalking:Std_Date	0.55	-17.13	16.821	0.958	0.514	-0.262	8.747	5.955
LocationWGL:Sound_TypeTalking:Std_Date	-10.608	-26.95	6.247	0.213	-10.58	-10.924	8.529	5.901

Table S4. Matched-model posterior estimates for reaction duration relative to the dove control. Posterior means (β) and 95% credible intervals (CrI) from the Bayesian mixed-effects model predicting the difference in reaction duration between each anthropogenic stimulus and its matched dove-call control. Positive values indicate longer durations for the anthropogenic stimulus compared to the matched dove trial. All sound-type CrIs overlap zero, demonstrating that anthropogenic stimuli did not reliably increase reaction duration relative to controls once contextual variables were matched. Random-effects estimates are reported for Giraffe ID, Playback event, and Playback exemplar.

Term	Mean (β)	Lower 95% CrI	Upper 95% CrI	*P*	Median	Mode	SE	MAD
(Intercept)	45.262	22.504	66.886	0	45.534	46.641	11.37	7.714
LocationFGR	-31.021	-59.53	0.252	0.046	-31.306	-32.43	15.21	10
LocationWGL	-38.775	-63.28	-12.655	0.003	-39.007	-40.611	12.96	8.729
Sound_TypeTalking	-9.149	-41.39	25.134	0.579	-9.056	-7.372	16.93	11.18
Sound_TypeVehicle	-30.066	-59.32	1.049	0.06	-30.308	-31.052	15.35	10.34
Std_Date	-10.927	-31.21	8.316	0.275	-11.011	-11.462	10.01	6.713
Sex_AgeAdult_M	2.834	-10	15.234	0.641	2.799	2.291	6.443	4.195
Sex_AgeSubAdult_F	0.382	-15.62	17.925	0.979	0.196	0.507	8.391	5.41
Sex_AgeSubAdult_M	3.231	-33.66	37.501	0.849	3.238	2.517	18.18	12.14
Std_grp_sz	-5.11	-11.58	1.483	0.127	-5.049	-4.727	3.355	2.253
d_poly_speaker_dist1	6.836	-67.83	77.168	0.865	6.24	6.035	37.44	24.96
d_poly_speaker_dist2	28.338	-35.02	89.006	0.38	27.75	23.867	31.9	21.44
Std_Wind_speed	2.332	-2.56	7.059	0.328	2.313	2.179	2.424	1.59
LocationFGR:Sound_TypeTalking	3.708	-32.59	40.106	0.832	3.865	2.38	18.56	12.26
LocationWGL:Sound_TypeTalking	3.577	-32.7	39.953	0.841	3.753	0.271	18.55	12.49
LocationFGR:Sound_TypeVehicle	18.304	-17.8	50.91	0.295	18.526	17.827	17.59	11.59
LocationWGL:Sound_TypeVehicle	27.546	-5.619	61.06	0.112	27.956	29.45	16.92	11.31
LocationFGR:Std_Date	23.823	0.244	44.788	0.039	23.944	23.991	11.43	7.854
LocationWGL:Std_Date	12.331	-7.799	33.545	0.241	12.49	13.729	10.53	7.124
Sound_TypeTalking:Std_Date	22.707	-3.619	48.242	0.09	22.878	22.779	13.26	8.977
Sound_TypeVehicle:Std_Date	16.39	-14.47	43.945	0.264	16.288	15.55	14.84	9.874
LocationFGR:Sound_TypeTalking:Std_Date	-26.098	-55.83	4.18	0.094	-26.381	-28.571	15.29	10.13
LocationWGL:Sound_TypeTalking:Std_Date	-18.863	-47.4	11.397	0.213	-18.964	-20.614	15	9.964
LocationFGR:Sound_TypeVehicle:Std_Date	-24.636	-58.21	7.758	0.139	-24.456	-25.141	16.85	11.15
LocationWGL:Sound_TypeVehicle:Std_Date	-12.741	-44.84	18.918	0.433	-12.462	-9.786	16.37	10.84
sigma	20.195	17.009	23.718	0	20.089	19.784	1.731	1.173
Sigma[Video_Number:(Intercept),(Intercept)]	205.56	0.008	373.31	0	201.21	200.94	98.09	65.55
Sigma[ID:(Intercept),(Intercept)]	91.577	0.001	210.17	0	77.962	60.973	63.18	33.04
Sigma(ID)	20.108	0	71.859	0	10.305	1.983	26.94	9.388

Table S5. Within-site pairwise contrasts for the raw reaction-intensity model. Posterior means (Average), medians, and 95% credible intervals (Lower–Upper) for all pairwise comparisons among stimulus types within each study site, based on the raw reaction-intensity model. Bold values indicate contrasts whose 95% credible intervals do not overlap zero, reflecting clear posterior support for a difference between stimulus types.

Location	Contrast	Average	Lower	Upper	p_value	adj_p_val
APGR	**Drone – Dove**	**4.266**	**2.679**	**5.803**	**0**	**0**
APGR	**Vehicle – Dove**	**5.294**	**3.495**	**7.084**	**0**	**0**
APGR	**Talking – Dove**	**1.884**	**0.281**	**3.505**	**0.023**	0.228
APGR	**Vehicle – Talking**	**3.41**	**1.222**	**5.589**	**0.003**	**0.045**
APGR	**Drone – Talking**	**2.382**	**0.419**	**4.355**	**0.015**	0.2
APGR	Vehicle – Drone	1.028	–1.005	3.178	0.337	1
FGR	**Drone – Dove**	**2.088**	**0.97**	**3.199**	**0.001**	**0.01**
FGR	**Talking – Dove**	**1.178**	**0.19**	**2.125**	**0.019**	0.207
FGR	Vehicle – Dove	1.065	–0.108	2.082	0.036	0.32
FGR	Drone – Vehicle	1.022	–0.233	2.266	0.116	0.812
FGR	Drone – Talking	0.91	–0.358	2.113	0.153	0.916
FGR	Talking – Vehicle	0.113	–1.019	1.302	0.837	1
WGL	**Talking – Dove**	**1.326**	**0.436**	**2.192**	**0.006**	0.078
WGL	**Drone – Dove**	**1.122**	**0.284**	**2.018**	**0.016**	0.2
WGL	Vehicle – Dove	0.948	0.062	1.884	0.046	0.371
WGL	Talking – Vehicle	0.378	–0.717	1.461	0.484	1
WGL	Talking – Drone	0.204	–0.865	1.251	0.702	1
WGL	Drone – Vehicle	0.174	–0.915	1.23	0.753	1

Table S6. Between-site pairwise contrasts for the raw reaction-intensity model. Posterior means (Average), medians, and 95% credible intervals (Lower-Upper) for between-site comparisons of reaction intensity across the three study locations (APGR, WGL, FGR), based on the raw reaction-intensity model. Positive values indicate stronger reactions at the first site in the contrast.

Location	Contrast	β (Mean)	Lower 95% CrI	Upper 95% CrI
APGR	Drone – Vehicle	30.066	–1.049	59.322
FGR	Drone – Vehicle	11.762	–4.698	29.279
APGR	Talking – Vehicle	20.917	–12.718	54.796
FGR	Talking – Vehicle	6.321	–9.071	21.913
WGL	Drone – Talking	5.571	–9.525	19.828
FGR	Drone – Talking	5.441	–11.036	21.915
APGR	Drone – Talking	9.149	–25.134	41.388
WGL	Vehicle – Talking	3.051	–11.685	17.981
WGL	Drone – Vehicle	2.521	–12.600	17.276

Table S7. Sex-age class contrasts for reaction intensity in the matched-model analysis. Posterior means (Average), medians, and 95% credible intervals (Lower–Upper) for pairwise contrasts among sex-age classes based on the matched-model analysis of reaction intensity. Positive values indicate higher reaction differences in the first sex-age class of the contrast. All credible intervals overlapped zero and adjusted p-values were high (adjusted p value > 0.60), indicating no supported differences among demographic groups.

Contrast	Average	Median	Lower	Upper	P value	adj. P value
Adult F – Adult M	0.9	0.909	-0.2	1.94	0.101	0.606
Sub-adult F – Adult M	0.86	0.85	-0.35	2.03	0.145	0.724
Adult F – Sub-adult M	2.929	2.917	-1.3	7.17	0.173	0.724
Sub-adult F – Sub-adult M	2.889	2.886	-1.31	7.46	0.197	0.724
Adult M – Sub-adult M	2.029	2.027	-2.38	6.23	0.36	0.724
Adult F – Sub-adult F	0.04	0.057	-1.24	1.28	0.931	0.931

Table S8. Within-site contrasts among anthropogenic stimuli from the matched-model analysis. Posterior means (Average), medians, and 95% credible intervals (Lower–Upper) for pairwise contrasts among anthropogenic sound types (drone, vehicle, people talking) within each study site (APGR, WGL, FGR). Positive values indicate higher reaction differences for the first stimulus of the contrast. Supported differences were detected only at APGR (where both vehicle and drone noise elicited stronger responses than human talking) whereas all contrasts within WGL and FGR showed wide, overlapping credible intervals. These results reflect greater stimulus discriminability at APGR and uniformly low responsiveness at the more human-exposed sites.

Location	Contrast	Average	Median	Lower	Upper	p_value	adj_p_val
APGR	Vehicle - Talking	4.628	4.602	1.18	8.07	0.01	0.09
APGR	Drone - Talking	3.326	3.351	0.064	6.41	0.044	0.349
WGL	Talking - Vehicle	0.785	0.784	-0.64	2.32	0.295	1
APGR	Vehicle - Drone	1.302	1.264	-1.58	4.29	0.39	1
WGL	Talking - Drone	0.402	0.4	-1.1	1.77	0.596	1
WGL	Drone - Vehicle	0.383	0.39	-1.11	1.85	0.598	1
FGR	Drone - Vehicle	0.244	0.259	-1.47	2.02	0.767	1
FGR	Drone - Talking	0.179	0.182	-1.45	1.87	0.826	1
FGR	Talking - Vehicle	0.065	0.086	-1.57	1.65	0.918	1

Table S9. Between-site contrasts in reaction intensity for each anthropogenic stimulus from the matched-model analysis. Posterior means (Average), medians, and 95% credible intervals (Lower–Upper) for contrasts comparing predicted reaction differences across study sites (APGR, WGL, FGR) for the three anthropogenic stimuli (drone, vehicle, people talking). Positive values indicate higher predicted reaction differences in the first site of the comparison. Strong differences were consistently observed between APGR and both WGL and FGR (particularly for vehicle and drone noise) whereas contrasts between WGL and FGR were small and uncertain. These between-site comparisons reinforce the population-level gradient in anthropogenic noise sensitivity inferred from the primary analyses.

Sound_Type	Contrast	Average	Median	Lower	Upper	P	adj_P
Vehicle	APGR - FGR	5.234	5.221	2.704	7.773	0	0.002
Vehicle	APGR - WGL	4.923	4.913	2.574	7.356	0	0.002
Drone	APGR - WGL	3.238	3.243	0.877	5.478	0.007	0.046
Drone	APGR - FGR	3.688	3.707	0.958	6.414	0.008	0.049
Talking	WGL - FGR	1.03	1.021	-0.632	2.624	0.207	1
Drone	WGL - FGR	0.449	0.459	-1.457	2.275	0.634	1
Vehicle	WGL - FGR	0.31	0.319	-1.597	1.976	0.726	1
Talking	WGL - APGR	0.49	0.496	-2.4	3.508	0.74	1
Talking	APGR - FGR	0.541	0.528	-2.691	3.752	0.744	1

Equation S1. Calculation of the 'behavioural reaction index'.

Let $I_{j=1,2,3} \in [0,12]$ be the intensity (0-12 score) of the first three observed behaviours after playback onset, and t_j its latency (s). The primary index is

$$R = \max_j I_j \times w(t_j), \qquad w(t) = \frac{1}{1 + e^{k(t-12)}}$$

We used a midpoint $t_0 = 12$ s and slope $k = 1$, which yields $w(9) \approx 0.95$ and $w(15) \approx 0.05$. Thus, reactions within ~9 s receive near-full weight and those after ~15 s contribute little.

Parameter choice:

Solving $0.95 = \frac{1}{1+e^{k(t-12)}}$ gives $k = 1.$, which also implies $w(15) \approx 0.05$. This parameterization emphasises rapid reactions while down-weighting later onsets.

Figure S1:

Supplementary Figure S1. Raw reaction intensity across age-sex classes and study sites. Reaction intensity (0–12 scale) of giraffes in response to four acoustic stimuli (dove [control], talking, drone, and vehicle) across age-sex classes and three study sites in the Free State Province, South Africa: Amanzi Private Game Reserve (APGR), Franklin Game Reserve (FGR), and Weltevreden Game Lodge (WGL). Raw data points (coloured by stimulus type) are shown together with model-derived expected values and 95% credible intervals from the raw reaction-intensity model. Age-sex class codes: Adult F = adult female, Adult M = adult male, SubAdult F = sub-adult female, SubAdult M = sub-adult male. This figure illustrates the distribution and variability of raw reaction-intensity responses prior to matched-model analyses.

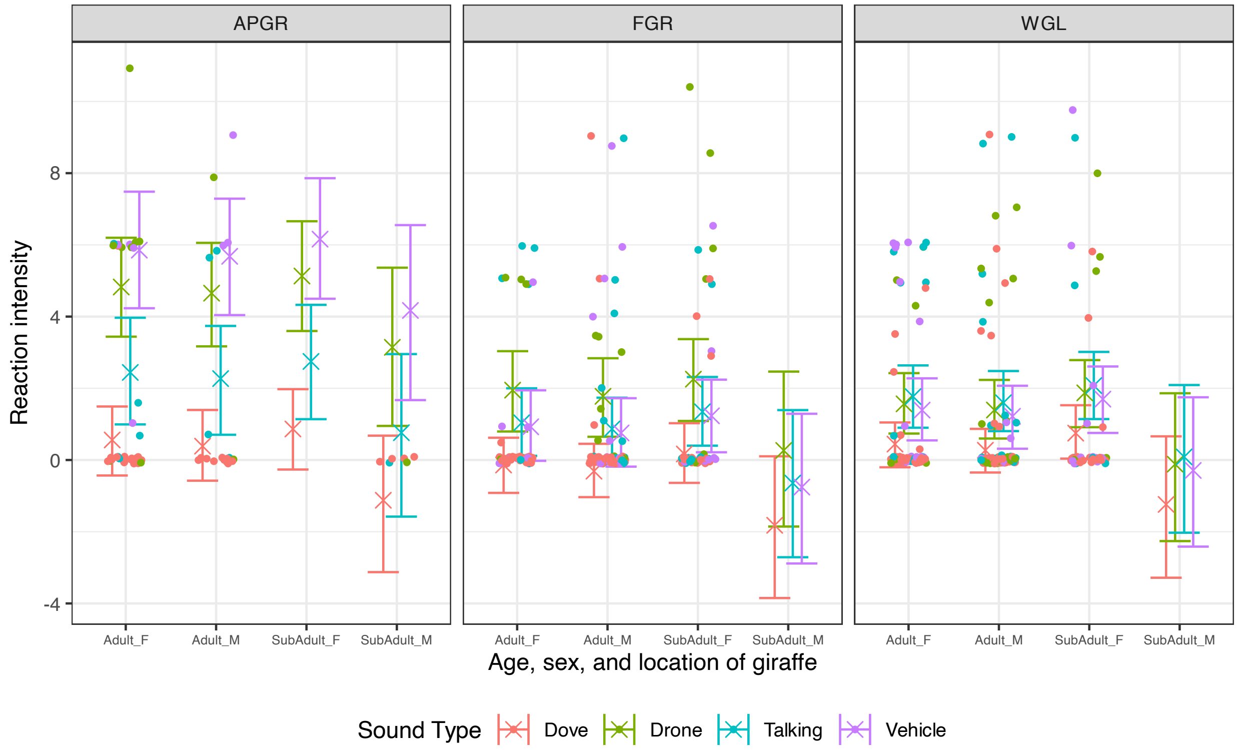

Supplement: Supplementary file 1 — Appendix S1: ece372763‐sup‐0001‐AppendixS1.zip. [file ECE3-15-e72763-s001.zip › ece372763-sup-0003-AppendixS3.pdf]
